# Combination chemotherapy in advanced gastrointestinal cancers: *ex vivo* sensitivity to gemcitabine and mitomycin C

**DOI:** 10.1038/sj.bjc.6601403

**Published:** 2003-12-09

**Authors:** P A Whitehouse, S J Mercer, L A Knight, F Di Nicolantonio, A O'Callaghan, I A Cree

**Affiliations:** 1 Department of Histopathology, Translational Oncology Research Centre, Queen Alexandra Hospital, Portsmouth PO6 3LY, UK; 2 Portsmouth Oncology Centre, St Mary's Hospital, Portsmouth PO3 6AD, UK

**Keywords:** colorectal cancer, oesophageal cancer, gemcitabine, mitomycin C, chemotherapy, ATP-TCA

## Abstract

Advanced or metastatic disease is common in both oesophagogastric and colorectal cancers, with poor 5-year survival despite palliative chemotherapy. We have investigated the sensitivity of gastrointestinal tumours to gemcitabine in combination with mitomycin C (GeM), using a modified *ex vivo* ATP-based tumour chemosensitivity assay (ATP-TCA). Tumour material from 41 colorectal and 22 oesophagogastric cancers were assessed. The GeM combination showed variable but definite activity in most of the samples tested. The results show that GeM achieves >95% inhibition at concentrations within the range achievable clinically in 60% of colorectal tumours (21 out of 35) and 38% of oesophagogastric tumours (five out of 13) tested. We did not identify any significant difference in sensitivity using concurrent or sequential exposure of tumour-derived cells to these two drugs. The results from this study suggest that GeM may be a useful combination in the treatment of advanced gastrointestinal malignancy.

Colorectal adenocarcinoma (CRC) is the second most common cause of cancer death in the western world. Despite potentially curable surgical treatment in 70–80%, half of all patients will die from metastatic disease, often within 5 years of diagnosis. Palliative chemotherapy may reduce the symptoms, extend survival from 6 to 12 months, and despite treatment-related adverse effects, often improve or at least maintain the quality of life (Nordic Gastrointestinal Tumour Adjuvant Therapy Group, 1992). In the UK, the current NICE guidance on chemotherapy in advanced colorectal disease (NICE, 2002) advocates the use of 5-fluorouracil (5FU) with folinic acid (FA) as first line therapy. This combination achieves response rates of 18–22% and overall survivals of 12–15 months (de Gramont *et al*, 2000; Maiello *et al*, 2000). Combinations using newer agents such as irinotecan and oxaliplatin achieve higher response rates of up to 50% and longer median overall survivals (Douillard *et al*, 2000; Saltz *et al*, 2000; Grothey *et al*, 2002). However, the newer agents, especially in combination with 5FU/FA, are associated with greater toxicity, which may be severe and dose-limiting.

Oesophageal and gastric cancers together account for 7% of all cancer-related deaths. Palliative treatment may be achieved with chemotherapy, radiotherapy or chemoradiation. A number of chemotherapy regimens are in use. Most are triple therapies incorporating 5FU and cisplatin with either epirubicin or paclitaxel. Response rates of 48–70% have been achieved (Kim *et al*, 1993; Ilson *et al*, 1998) with a 2-year survival of 13.5% (Findlay *et al*, 1994); however, in general, the responses are often short lived and these treatments are associated with varying degrees of toxicity.

Recently, we investigated the chemosensitivity of colorectal and oesophagogastric adenocarcinomas (Mercer *et al*, 2003; Whitehouse *et al*, 2003) using a modified *ex vivo* ATP-based tumour chemosensitivity assay (ATP-TCA) (Andreotti *et al*, 1995; Cree *et al*, 1996). We demonstrated considerable differences in sensitivity between individual tumours of both tumour types. Mitomycin C (MMC) has been used in the treatment of gastrointestinal tumours for many years, although it is not now the most commonly used drug due to serious pulmonary, renal and haematological toxicities, which tend to occur with overdosage. It remains useful in the treatment of metastatic gastrointestinal tumours, usually in combination with 5-FA. Gemcitabine is licensed for use in pancreatic and non-small-cell lung cancers. It has also shown preclinical and clinical activity in several other solid tumours, including ovarian, head and neck and breast cancers (Carmichael *et al*, 1995; Markman, 2002). However, phase I/II trials of single-agent gemcitabine have not demonstrated any activity in advanced colorectal and gastric cancers (Moore *et al*, 1992; Christman *et al*, 1994; Mani *et al*, 1998). We therefore wished to test this combination on further gastrointestinal tumour samples and investigate any schedule dependency.

## MATERIALS AND METHODS

### Tumours

Material from 41 colorectal and 22 oesophagogastric tumours was tested. All the colorectal tumours were previously untreated. Six of the oesophagogastric tumours had received neoadjuvant chemotherapy with epirubicin+cisplatin+5-FA (ECF). The median age of patients undergoing colorectal resection was 70 years (range 39–86) and for oesophagogastric resection was 69 years (range 39–87). The local ethics committee approval for the use of tissue or cells not required for diagnosis was obtained, and informed consent gained from all patients.

### ATP-tumour chemosensitivity assay

The ATP-tumour chemosensitivity assay (ATP-TCA) was performed as previously described (Andreotti *et al*, 1995; Cree, 1998). Tumour samples were transported to the laboratory in transport medium consisting of Dulbecco's modified Eagle's medium (Sigma, Poole, UK; D5671), minced and dissociated overnight in collagenase (Sigma; C8051). The concentration of collagenase used was 0.75 mg ml^−1^ for oesophagogastric samples and 1.5 mg ml^−1^ for colorectal samples. If necessary, the samples were purified using Ficoll-hypaque density centrifugation (Sigma; 1077-1) to remove red blood cells and cell debris. The remaining cells were resuspended in antibiotic containing serum-free complete assay medium (CAM) (DCS Innovative Diagnostik Systeme, Hamburg, Germany) at 200 000 cells ml^−1^. Additional amphotericin B (2.5 *μ*g ml^−1^) was added to the CAM for the oesophagogastric samples, and amphotericin B (2.5 *μ*g ml^−1^) and metronidazole (1 *μ*g ml^−1^) were added to the CAM for the colorectal samples, as previously described (Whitehouse *et al*, 2003). 96-well polypropylene plates (Corning-Costar, High Wycombe, UK) were prepared with 100 *μ*l of CAM, to which the drugs were added at six concentrations in triplicate. Two internal controls were included in each plate: a maximum inhibitor (MI) that kills all the cells resulting in a zero ATP count, and a medium only (MO) without any drugs. After 6 days incubation at 37°C with 5% CO_2_, the cells were lysed with a detergent-based tumour cell extraction reagent (DCS Innovative Diagnostik Systeme). A volume of 50 *μ*l from each well was transferred to the wells of a 96-well white plate (Thermo Life Sciences, Basingstoke, UK), to which 50 *μ*l of luciferin-luciferase counting reagent (DCS Innovative Diagnostik Systeme) was added. The ATP content of each well was quantified by the amount of light produced in a microplate luminometer (Bethold MPLX). The results are expressed as the percent inhibition achieved at each concentration tested, calculated as: % inhibition=1−(test−MI)/(MO−MI) × 100.

### Drugs

Mitomycin C (Kyowa, London, UK) and Gemcitabine (Eli Lilly, Basingstoke, UK) were obtained as vials for injection, and made up according to the manufacturer's instructions. Both MMC and gemcitabine were stored as aliquots at −20°C (Hunter *et al*, 1994). The 100% test drug concentrations (TDCs) used were calculated from pharmacokinetic data to approximate concentrations clinically achievable in the patient (Andreotti *et al*, 1995). While this has inevitable inaccuracies, the 100% TDC of MMC was 0.7 *μ*g ml^−1^ (2.0 *μ*M) and the 100% TDC of gemcitabine was 12.5 *μ*g ml^−1^ (40 *μ*M). Drug dilutions were prepared in the plates from freshly made up 800% TDC drug solutions. Combinations of drugs were made by adding both drugs, each at its 800% TDC. In combinations tested in the assay, drugs are present at decreasing concentrations in a constant ratio.

Sequential studies were performed by testing (i) MMC and gemcitabine, both added at 0 h; (ii) MMC at 0 h followed by the addition of gemcitabine at 6 h; (iii) MMC at 0 h and gemcitabine at 24 h; and (iv) gemcitabine at 0 h with MMC added at 24 h.

### Data analysis

Data from each assay were transferred directly from the luminometer to an Excel 2000 spreadsheet (Microsoft). A number of indices of efficacy can be calculated from the data, including the IC_90_. The natural logarithmic sum index (Index_SUM_), calculated by summing the percentage inhibition at each concentration, has been found to allow the best comparison of responses between samples (Hunter *et al*, 1993). In addition, the area under the concentration–inhibition curve (Index_AUC_) and the percentage of tumours achieving 95% inhibition have been calculated. Combination effects were assessed using the method established by Poch *et al* (1995), as previously used with the ATP-TCA (Kurbacher *et al*, 1997). This is used in preference to the Chou and Talalay method, because it is better able to deal with drugs which produce a shallow dose–response curve (Chou and Talalay, 1984). However, we have also performed a Chou and Talalay analysis, where the combination index (CI) was determined at 90% cell death, and was defined as follows:


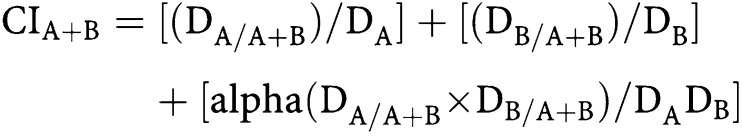


where CI_A+B_=CI for a fixed effect (F=90%) for the combination of cytotoxic A and cytotoxic B; *D*_A/A+B_=concentration of cytotoxic A in the combination A+B, giving an effect F; *D*_B/A+B_=concentration of cytotoxic B in the combination A+B, giving an effect F; *D*_A_=concentration of cytotoxic A alone, giving an effect F; *D*_B_=concentration of cytotoxic B alone, giving an effect F. alpha=parameter with value 0 when A and B are mutually exclusive, and 1 when A and B are mutually nonexclusive.

The combination index indicated: synergism <0.8; additivity >0.8 and <1.2; antagonism <1.2; slight synergistic and additive cytotoxic activity for values of 0.8 and 1.2, respectively.

## RESULTS

Evaluable results were obtained from 60 out of 63 tumours, giving an evaluability rate of 95%. Three tumours were not evaluable due to contamination of the cell culture. Despite the addition of extra antibiotics during specimen preparation, contamination is a problem with such tissue and these samples are technically challenging (Whitehouse *et al*, 2003).

**Figure 1:**
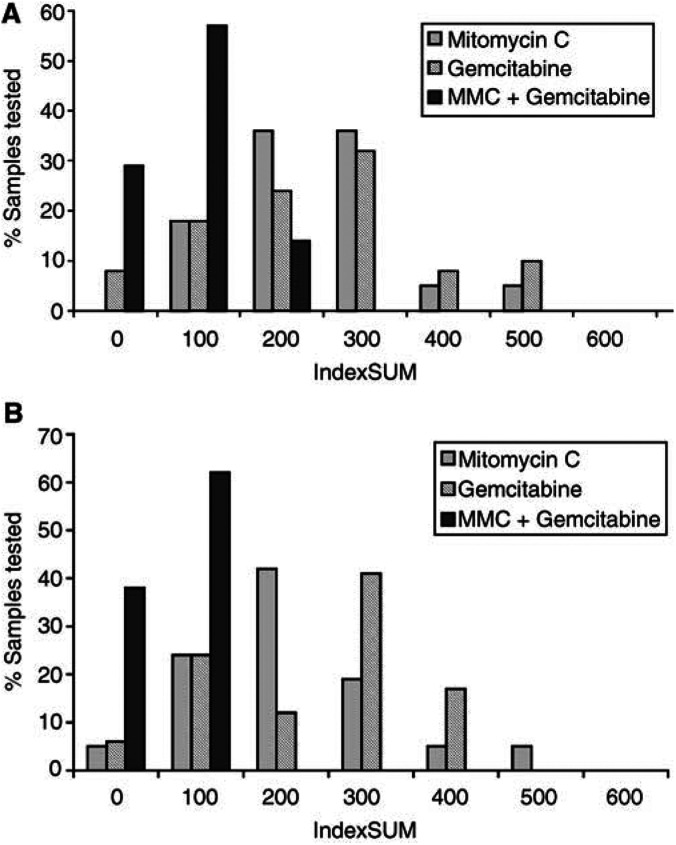
Summary of the heterogeneity of activity in the ATP-TCA to MMC, gemcitabine, and the combination (GeM) in (**A**) colorectal cancer (*n*=39), and (**B**) oesophagogastric cancer (*n*=21). The IndexSUM is a parameter describing the concentration–inhibition curve for each drug, or combination in a single number. Using the IndexSUM <300 to indicate sensitivity, GeM is clearly more active than either single agent.

The single-agent results are shown in Figure 1

and Table 1

**Table 1:** Summary of sensitivity data (using an arbitrary threshold of sensitivity defined as a IndexSUM<300 for six concentrations used)

**Drug**	**No. assessed**	**No. sensitive**	**Sensitivity (%) (Index <300)**	**>95% Inhibition (%)**
*Oesophagogastric cancer*				
Mitomycin C	21	15	71	14 (three out of 21)
Gemcitabine	17	7	42	6 (one out of 17)
Mitomycin C+Gemcitabine	13	13	100	38 (five out of 13)
				
*Colorectal cancer*				
Mitomycin C	39	21	54	10 (four out of 39)
Gemcitabine	38	19	50	3 (one out of 38)
Mitomycin C+gemcitabine	35	35	100	60 (21 out of 35)

. There is considerable heterogeneity between tumours for these two drugs, with sensitivity to MMC in 71% of the oesophagogastric tumours (15 out of 21) and 59% of the colorectal tumours (21 out of 39). For comparison between drugs and tumours, an Index_SUM_ of <300, representing an average 50% inhibition across all concentrations tested, has been used to indicate sensitivity, as previously published (Hunter *et al*, 1993; Cree *et al*, 1999). Despite these apparently encouraging results, MMC alone achieves >95% inhibition at clinically achievable concentrations in just 14% of oesophagogastric tumours (three out of 21) and 10% of colorectal tumours (four out of 39) tested. Gemcitabine alone is active on the basis of an IndexSUM <300 in 42% of oesophagogastric tumours and 50% of colorectal tumours. However, it tends to have a very shallow dose–response curve and only rarely produced >95% inhibition at clinically achievable concentrations: 6% of oesophagogastric tumours (one out of 17) and 3% of colorectal tumours (one out of 38).

In contrast to the single-agent results, gemcitabine in combination with MMC (GeM) achieves >95% inhibition at clinically achievable concentrations in 60% of colorectal tumours (21 out of 35) and 38% of oesophagogastric tumours (five out of 13) tested (Table 1). The use of an Index_SUM_ <300 threshold for combinations tends to overstate the sensitivity, and it is not surprising that 100% of the tumours tested reached this threshold for sensitivity, as applied to single agents. Even when the Index_SUM_ is decreased to <200, GeM is active in 100 and 89% of oesophagogastric and colorectal tumours, respectively. Figure 1 shows this as a shift in activity towards the lower concentrations tested. In oesophageal tumours, the addition of gemcitabine decreased the MMC IC_90_ from 3.89 to 0.86 *μ*M, and for colorectal tumours the MMC IC_90_ was decreased from 3.73 to 0.96 *μ*M. Using the Chou and Talalay method, this equates for oesophageal cancers to a CI90 of 0.41 (synergism) and for colorectal cancers to a CI90 of 0.44 (synergism).

**Figure 2:**
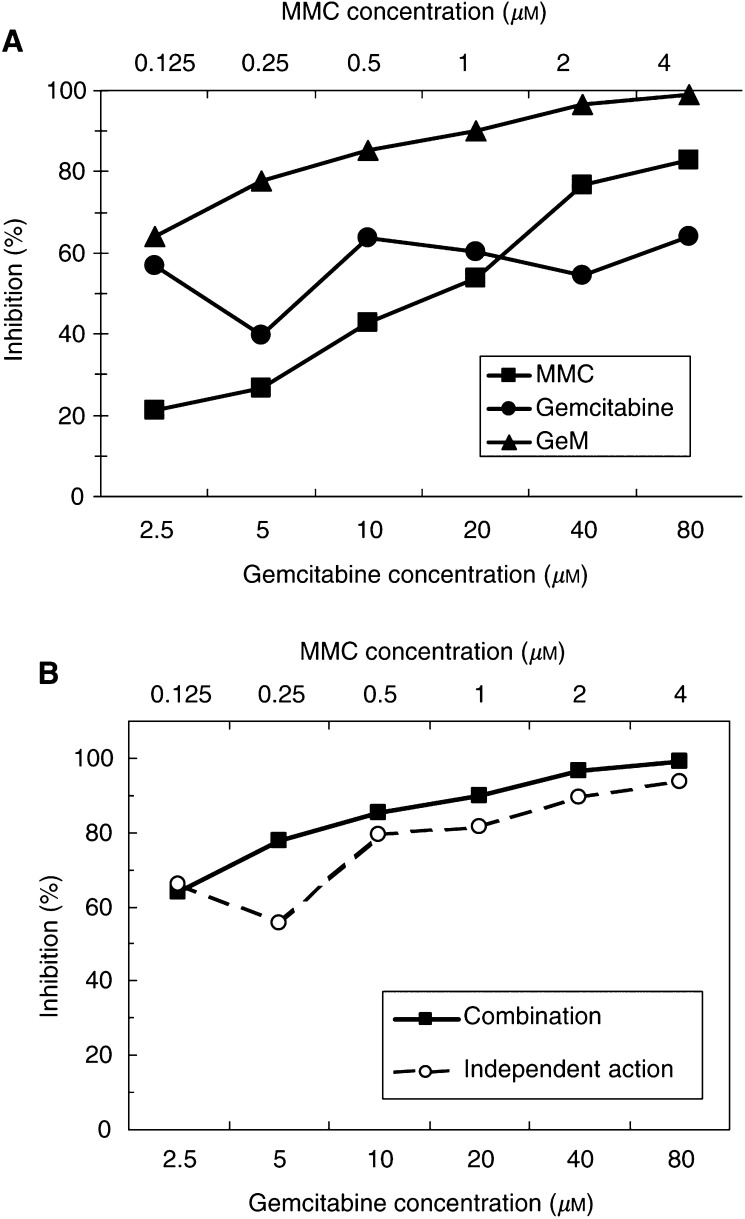
(**A**) Inhibition–concentration curve in a colorectal tumour sample. (**B**) By the method of Poch *et al*. (1995), the observed effect of the combination at each concentration is greater than the expected effect (independent action).

The CRC example shown in Figure 2A

shows the advantage of the GeM combination over the individual agents in terms of inhibition. When analysed by the method of Poch *et al* (1995), by which the observed effect at each concentration tested is compared with that expected, the effect is greater than additive (Figure 2B).

**Figure 3:**
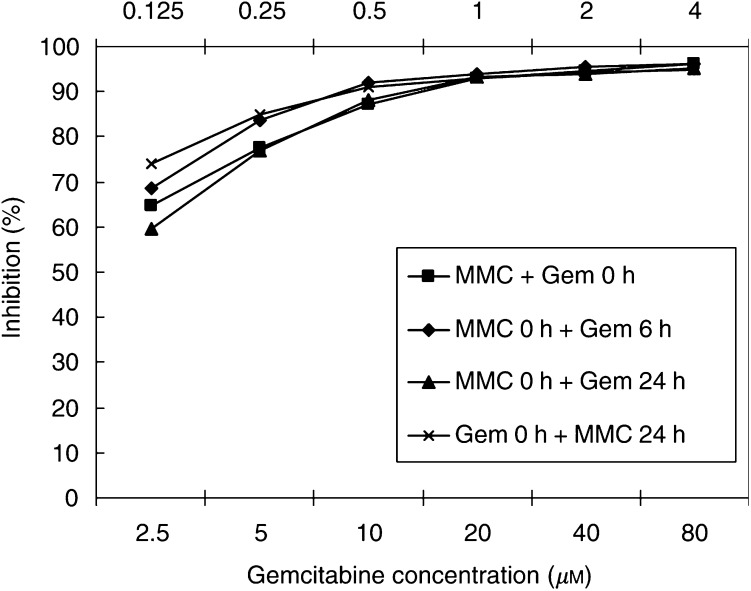
Schedule studies of gemcitabine in combination with MMC. No schedule dependency has been demonstrated.

Schedule experiments of gemcitabine and MMC are shown in Figure 3

. These experiments were performed, in at least triplicate, by adding 6.25–200% TDC of gemcitabine to 6.25–200% TDC of MMC at 0, 6 and 24 h. Mitomycin C was also added to gemcitabine at 24 h. There was no apparent difference in inhibition between the different schedules (93.84–95.84% tumour growth inhibition at 100% TDC), although gemcitabine added to MMC at 24 h did produce the lowest growth inhibition of 93.84%).

## DISCUSSION

These results show that, *ex vivo*, the combination of gemcitabine and MMC (GeM) is more effective than the single agents alone, when tested on gastrointestinal tumours. Our previous studies have shown this to be the most active combination in the assay, when tested alongside standard treatment regimens (Mercer *et al*, 2003; Whitehouse *et al*, 2003), with 38% of oesophagogastric tumours and 60% of colorectal tumours tested, showing >95% inhibition in the *ex vivo* ATP-TCA. We believe the 95% inhibition figure to be important, as this is the level of tumour cell kill required for most logarithmic kill models to show effects (Goldie and Coldman, 1979). While these data may not of course translate into clinical efficacy of similar magnitude, it does provide a basis for the clinical investigation of this combination in gastrointestinal tumours. The difference in sensitivity between the two tumours may be related to tumour type, to the proportion of previously treated oesophageal cancer samples included in the study, or to the necessary inclusion of metronidazole in the cell cultures from colorectal tumours. In a previous study (Whitehouse *et al*, 2003), we tested the antibiotics at varying concentrations in combination with chemotherapeutic agents against cell lines and tumour samples, and were unable to show any additional toxicity at the concentrations used in this study. We believe that the effect of previous treatment may be a major factor in this unexpected difference between these two tumour types.

Mitomycin C has been used in the treatment of gastrointestinal malignancies for over 30 years, and has been shown to be relatively safe and effective (Chester *et al*, 2000), although very rarely patients may develop the haemolytic–uraemic syndrome, usually at very high doses (Catalano *et al*, 2002; Gundappa *et al*, 2002). Pulmonary and renal toxicities are also a problem in some patients, again usually at high cumulative doses. It is a DNA-damaging drug, inhibiting DNA synthesis by crosslinking adenosine and guanine under anaerobic conditions (Spanswick *et al*, 1998). Single-agent MMC has produced response rates of up to 23% in colorectal cancer (Moertel *et al*, 1968; Moore *et al*, 1968). The combination of MMC and 5FU has shown synergistic growth inhibition of cell lines (Sartorelli and Booth, 1965), including colorectal cancer cell lines (Russello *et al*, 1989). A randomised controlled trial in colorectal cancer found that MMC in combination with protracted venous infusion (PVI) 5FU increased the response rates to 54%, but with no benefit to overall and 1-year survival (Ross *et al*, 1997). A further phase III study confirmed an improved response rate with a survival benefit at 2 years (Price *et al*, 1999). Mitomycin C has been used in combination therapy of oesophagogastric cancers. Although initial response rates of 40% were quoted for treatment with FAM (5FU, doxorubicin and MMC) (MacDonald *et al*, 1979), no benefit has been demonstrated from the addition of MMC to 5FU (Tebbutt *et al*, 2002). MCF (MMC, cisplatin and 5FU) has no survival advantages over standard treatment with ECF (epirubicin, cisplatin, 5FU) (Ross *et al*, 2002).

Gemcitabine is an antimetabolite cytidine analogue with a number of mechanisms of cytotoxicity. Intracellular phosphorylation to its active metabolite results in (a) prevention of DNA synthesis by inhibiting DNA polymerases and by competing with deoxycytidine triphosphate, (b) inhibition of ribonucleotide reductase, depleting deoxynucleotide pools and favouring incorporation of gemcitabine into DNA, (c) incorporation into DNA, decreasing the accuracy of DNA replication and repair, and (d) incorporation into RNA. It should be noted that the effect of gemcitabine on ribonucleotide reductase could affect ATP levels, possibly as part of its cytostatic effect, though gemcitabine is inactive against many solid tumours (Neale *et al*, 1999) and this does not seem to be a problem for its use in the assay.

Gemcitabine, which is cell cycle specific, and well tolerated clinically, is an attractive drug for use in combination with DNA-damaging agents (Huang *et al*, 1991); particularly it has been shown to modulate the activity of a wide range of DNA-damaging agents, including platinum (Peters *et al*, 1995; Sandler *et al*, 2000) and alkylating agents (Neale *et al*, 1999). We have not investigated the exact mechanism of modulation of MMC sensitivity with gemcitabine, but this may be due to inhibition of repair of alkylating agent-induced DNA adducts, an increase in DNA double-strand breaks or changes in dNTP pools. Studies of gemcitabine in combination with cisplatin are based on this mechanism of action (Cardenal *et al*, 1999). This study raises the question as to whether other alkylating agents might be effective in combination with gemcitabine in gastrointestinal cancer.

There are very few *in vitro* studies of gemcitabine and MMC. The combination was found to be synergistic after 4 h on a Lewis lung cancer cell line, without any increase in DNA double-strand breaks (van Moorsel *et al*, 1997). Similarly, MMC and gemcitabine had a synergistic effect, when administered concurrently, but not sequentially, on the HT29 human colon cancer cell line (Aung *et al*, 2000), suggesting that gemcitabine could be beneficial in the treatment of cancers sensitive to MMC.

Clinical studies are also few in number: intravenous and intra-arterial locoregional treatment with MMC and gemcitabine has been found to be highly effective with improved response rates in pancreatic cancer (Klapdor *et al*, 2000). This drug combination (median total dose MMC 32 mg m^−2^) has been administered together with radiotherapy with tolerable toxicity (Korneck *et al*, 2001).

In this study, we have not demonstrated any schedule-specific alterations in chemosensitivity; there were no protective effects on the cells of prior addition of gemcitabine as we have previously reported for treosulfan+gemcitabine (Neale *et al*, 1999). It should be noted that the nature of the assay means that, in all experiments, the cells were exposed to the combination for at least 5 days. However, a study of MMC and gemcitabine on the HT29 human colon cancer cell line showed simultaneous exposure to be necessary to demonstrate synergism (Aung *et al*, 2000). The effect of gemcitabine in combination with other alkylating and platinum agents has also been shown to be time-dependent (Braakhuis *et al*, 1995; Neale *et al*, 1999; van Moorsel *et al*, 2000). Since simultaneous administration is generally preferable to patients and oncology units, it would probably be reasonable to give both drugs together in future clinical studies.

These results demonstrate that gemcitabine+MMC (GeM) is effective *ex vivo* against tumour-derived cells from both oesophageal and CRCs. There is little evidence of schedule dependency and simultaneous administration should be feasible. These results have encouraged us to explore the GeM regimen further in a phase I/II clinical trial to establish its safety and efficacy in metastatic gastrointestinal cancer.
